# Development of Novel Genomic Simple Sequence Repeat (g-SSR) Markers and Their Validation for Genetic Diversity Analyses in Kalmegh [*Andrographis paniculata* (Burm. F.) Nees]

**DOI:** 10.3390/plants9121734

**Published:** 2020-12-09

**Authors:** Ramesh Kumar, Chavlesh Kumar, Ritu Paliwal, Debjani Roy Choudhury, Isha Singh, Ashok Kumar, Abha Kumari, Rakesh Singh

**Affiliations:** 1 Division of Genomic Resources, ICAR-National Bureau of Plant Genetic Resources, New Delhi 110012, India; ramesh.manglesha@gmail.com (R.K.); ritu4paliwal@gmail.com (R.P.); roydebj@gmail.com (D.R.C.); 2 Amity Institute of Biotechnology, Amity University Uttar Pradesh, Noida 201313, Uttar Pradesh, India; akumari@amity.edu; 3 Division of Fruits and Horticultural Technology, ICAR-Indian Agricultural Research Institute, New Delhi 110012, India; ckfruits2016@gmail.com; 4 Division of Plant Physiology, ICAR-Indian Agricultural Research Institute, New Delhi 110012, India; isha.singh2196@gmail.com; 5 School of Biomolecular and Biomedical Sciences, University College of Dublin, D04V1W8 Dublin, Ireland; 6 Division of Germplasm Evaluation, ICAR-National Bureau of Plant Genetic Resources, New Delhi 110012, India; Ashok.Kumar28@icar.gov.in

**Keywords:** microsatellite, AMOVA, PCoA, genetic diversity, genomic resource

## Abstract

Kalmegh (*Andrographis paniculata* (Burm. F.) Nees) is one of the most important medicinal plants and has been widely explored as traditional medicine. To exploit its natural genetic diversity and initiations of molecular breeding to develop novel cultivars or varieties, developments of genomic resources are essential. Four microsatellite-enriched genomic libraries—(CT)_14_, (GT)_12_, (AG)_15_ and (AAC)_8_—were constructed using the genomic DNA of *A. paniculata*. Initially, 183 recombinant colonies were screened for the presence of CT, GT, AG, and AAC microsatellite repeats, out of which 47 clones found positive for the desired simple sequence repeats (SSRs). It was found that few colonies had more than one desirable SSR. Thus, a sum of 67 SSRs were designed and synthesized for their validation among 42 *A. paniculata* accessions. Out of the 67 SSRs used for genotyping, only 41 were found to be polymorphic. The developed set of g-SSR markers showed substantial genetic variability among the selected *A. paniculata* accessions, with an average polymorphic information content (PIC) value of 0.32. Neighbor-joining tree analysis, population structure analysis, analysis of molecular variance (AMOVA), and principal coordinate analysis (PCoA) illustrated the considerable genetic diversity among them. The novel g-SSR markers developed in the present study could be important genomic resources for future applications in *A. paniculata*.

## 1. Introduction

The Kalmegh is an important medicinal crop species that is botanically known as *Andrographis paniculate* (Burm. F.) Nees and belongs to the family Acanthaceae [1]. This is an annual herb, about a meter in height, and bitter in taste, like *Neem* [2]; thus, Kalmegh is often called the king of bitter plants [3]. The plant is diploid (2n = 2x = 50) and found in both cultivated and wild forms in India [4]. *A. paniculata* is widely distributed in Asian countries like India, Sri Lanka, and China [5]. Andrographolide is one of the major bitter-tasting secondary metabolites derived from Kalmegh, a major bioactive substance responsible for the therapeutic interest [6]. This crop species is widely used as a traditional medicine in different parts of the world due to its versatile biological properties like immune-stimulatory [7], hepatoprotection [8], antibacterial [9], antimalarial [10], antithrombotic [11], antitumor [12], and anti-inflammatory [13]. Thus, this crop species has been used to treat various human diseases such as diabetes, hepatitis, leprosy, HIV, bronchitis, hypertension, cancer, and kidney disorders [14]. Currently, Kalmegh has been declared by the Indian National Medicinal Plants Board to be one of the most prioritized plant species among medicinal crop species for the exploitation of its potential use in human disease control and therapeutics [15].

Harnessing the genetic variability and development of the superior Kalmegh varieties with enhanced medicinal value is one of the prime objectives among researchers. Hence, genetic characterization of germplasm is one of the essential and primary steps in crop breeding programs. An in-depth characterization of germplasm allows effective selection of diverse parents in varietal improvement, besides helping efficient germplasm management in any crop species. Since distinguishing the genotypes based on morphological traits is time-consuming [16], the use of molecular markers can overcome its limitations. In the past, molecular markers like RAPD (randomly amplified polymorphic DNA) [3,4,17], AFLP (amplified fragment length polymorphism) [17], ISSR (inter simple sequence repeats), SCoT (start codon targeted polymorphism), and CBDP (CAAT box-derived polymorphism) [4] have been used for the genetic characterization of *A. paniculate* accessions. However, these dominant marker systems have low reproducibility and low consistency [18], which is a significant impediment for their further utilization in *A. paniculate* genomics and molecular breeding. Moreover, these dominant marker systems, namely, microsatellite or simple sequence repeat (SSR) markers, are one of the most preferred marker systems in studies of plant breeding and genetics due to the abundance in genomes, codominant natures, high reproducibility, multiallelic traits, and high transferability across the species [19]. Only the plastid genome sequence of *A. paniculata* has been carried out, and the whole genome of this crop is still not sequenced. It is, therefore, essential to develop microsatellite markers in *A. paniculata*.

Several methods have been employed to develop SSR markers in different plant species, and the microsatellite enrichment method is considered one of the most robust, reproducible, and cost-effective techniques [20]. This technique has been exploited for the development of SSR markers in several plant species including medicinal crops *viz*., *Paeonia lactiflora* [21], *Centella asiatica* [22], *Tinospora cordifolia* [23], and *Bauhinia strychnifolia* [24]. Therefore, the development of novel SSR markers was undertaken in *A. paniculata* using the microsatellite enrichment technique. The efficacy and informativeness of the developed SSRs were validated through genetic diversity studies among the *A. paniculata* accessions collected from different Indian states and maintained at ICAR-NBPGR, New Delhi, in the National Gene Bank.

## 2. Materials and Methods

### 2.1. Plant Materials

**Figure 1:**
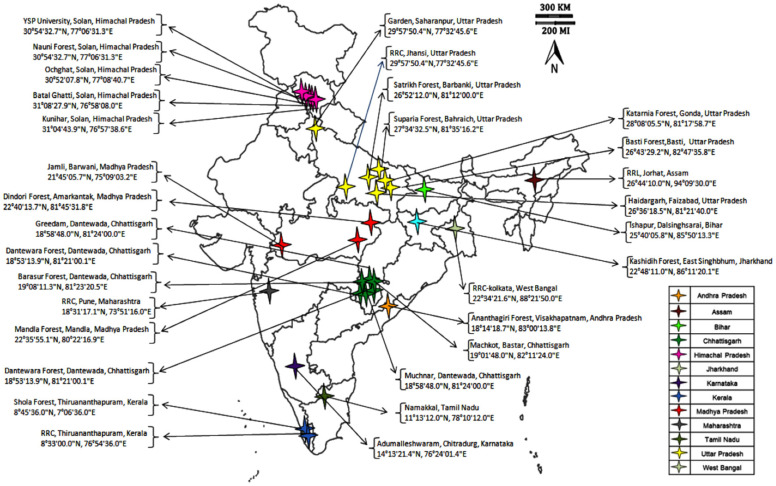
*Andrographis paniculata* accessions collected from different geographical regions of India.

Seeds of 42 *A. paniculata* accessions were collected from the National Gene Bank ICAR-NBPGR, New Delhi, which were earlier collected from different geographical regions of Indian states (Figure 1). The collected seeds were sown in the experimental field at Issapur Research Farm (situated at 28.24 N latitude and 76.50 E longitude and an elevation of 190.7 m above sea level). The details on the *A. paniculata* accessions and their collection places are given in Table 1.

### 2.2. Plant Genomic DNA (gDNA) Isolation

The young and healthy leaves of each accession were collected at 45 days after sowing, snap-frozen, and stored at −80 °C for further use. The genomic DNA was isolated, following the CTAB method described by Doyle and Doyle [25], with minor alterations. Since *A. paniculata* is rich in phenolic compounds, 3% polyvinylpyrrolidone (PVP) was also used to reduce the phenolics and facilitate quality gDNA extraction. To eliminate RNA contamination, 2.5 U of RNaseA enzyme (Himedia, West Chester, PA, USA) was used. The DNA quality was evaluated on 0.8% agarose gel, and its concentration was determined using a NanoDrop instrument (Thermo Fisher Scientific, Waltham, MA, USA).

### 2.3. Construction of the Microsatellite-Enriched Library

The microsatellite-enriched genomic libraries in *A. paniculata* were developed using the altered biotin-capture method, as suggested by Fischer and Bachmann [26]. The gDNA of *A. paniculata* accession IC111291 (1000 ng) was digested using the restriction enzyme *Sau3A1* (New England Bio Labs, Knowl Piece Wilbury Way Hitchin UK) by incubation at 37 °C for 2 h and, later, inactivating it at 65 °C for 20 min. The restriction digestion of gDNA and the adapter-ligation of DNA were done as suggested by Bloor et al. [27].

SSR-containing DNA fragments were acquired by hybridization reaction of an adaptor-attached DNA fragment with prewashed (1X washing buffer and 2X washing buffer, respectively) streptavidin-coated magnetic beads and 3′-biotinylated oligonucleotide probes ((CT)_14_, (GT)_12_, (AG)_15_ and (AAC)_8_) at 60 °C for 30 min, with frequent shaking at 5 min intervals in 6X SSC buffer(Saline Sodium Citrate buffer). After the hybridization reaction, the magnetic beads were separated using the magnetic stand and the incubation of hybridization products in 2X SSC and 1XSSC, respectively, followed by final boiling at 95 °C for 15 min in TE buffer (Tris-EDTA). The concentration of enriched DNA was enhanced by performing a PCR reaction [27]. Thereafter, the PCR-amplified products were ligated into pCR 2.1 Cloning Vector (Thermo Fisher Scientific, Waltham, MA, USA) overnight at 16 °C and transformed into *E. coli* (*Escherichia coli*) DH5α competent cells. As per the X-gal/IPTG (5-Bromo-4-chloro-3-indolyl β-D-galactopyranoside (X-gal)/ Isopropyl-β-D-thiogalactoside (IPTG))selection method, a total of 183 white colonies were screened, from which 119 positive clones were selected while performing colony PCR (using M13 universal primer). The plasmid DNA from selected positive clones was extracted using a plasmid isolation kit (Zymo Research, Irvine, California, USA). The plasmids were subsequently sequenced, along with M13 primers, using Sanger’s dideoxy sequencing approach (Macrogen Inc., Seoul, Korea).

### 2.4. SSR Finding and Primer Designing

After the trimming of vector sequences, the sequencing results of positive clones were searched for desirable microsatellite repeats using an online SSR finder tool (http://www.csufresno.edu/ssrfinder/). The microsatellite primer pairs were designed based on the sequences flanking the SSR motifs using an online tool, Primer 3.0 input version 0.4.0 (http://bioinfo.ut.ee/primer3-0.4.0/). Finally, primer pairs were designed in the range of 18–25 nucleotides having an amplicon size ranging from 100 to 500 base pairs.

### 2.5. Polymerase Chain Reaction

A set of 67 developed SSR primers were selected for genetic diversity analysis, and these primers were amplified on 42 *A. paniculata* accessions, out of which 41 were found reproducible and polymorphic. The gDNA of selected 42 accessions was isolated, and its final working concentrations were kept at 10 ng/µL. The PCR reaction was performed in the total volume of 25 µL containing 7 µL gDNA (70 ng) as a template, 2.5 µL of 10X Dream*Taq* buffer, 3 µL of 2.5 mM MgCl2, 2.5 µL of 2.5 mM dNTPs, 0.8 µL of each primer (10 nmol), and 0.4 µL of Dream*Taq* DNA polymerase enzyme (Thermo Scientific, Waltham, MA, USA), with 8.8 µL Milli-Q water added to make the final volume. PCR amplification was performed in a thermocycler (Gstorm, Essex, England) using following the PCR cycle: initial denaturation at 94 °C for 4 min, followed by 36 cycles of denaturation at 94 °C for the 30 s, annealing temperature (standardize by gradient PCR) for 45 s, extension at 72 °C for 2 min, and a final extension at 72 °C for 10 min. The PCR products were checked on 4% metaphor agarose gel (Lonza, Rockland, ME, USA) for 4 h at a constant supply of 120 V, and gel images were captured using a gel documentation system (Alpha Imager^®^, Bengaluru, Karnataka, India).

### 2.6. Data Scoring and Statistical Analyses

The amplified PCR products of each primer pair among the *A. paniculata* accessions were scored using PyElph 1.4 [28]. The genetic diversity statistics viz. the dominant allele frequency, gene diversity, heterozygosity, and polymorphic information content (PIC) were calculated using Power Marker 3.5 [29]. An unrooted neighbor-joining (N-J) tree was generated, and the genetic distances between the *A. paniculata* accessions were also estimated using Power Marker 3.5 software [29]. Model-based population structure analysis was performed using STRUCTURE software version 2.3.4 [30]; the software was run multiple times by setting k (the number of populations) from 2 to 10, the length of burn-in period and number Markov Chain Monte Carlo (MCMC) replications were set at 100,000 for each run for all 42 genotypes to evaluate the number of populations [31]. An online tool, Structure Harvester (http://taylor0.biology.ucla.edu), was used to calculate the most probable genetic population groups of the studied *A. paniculate* accessions. Principal coordinate analysis (PCoA), analysis of molecular variance (AMOVA), and the Mantel test were done using the program GenAlEx 6.5 [32].

## 3. Results and Discussion

### 3.1. Development of SSR Markers from Enriched Genomic Libraries

**Figure 2:**
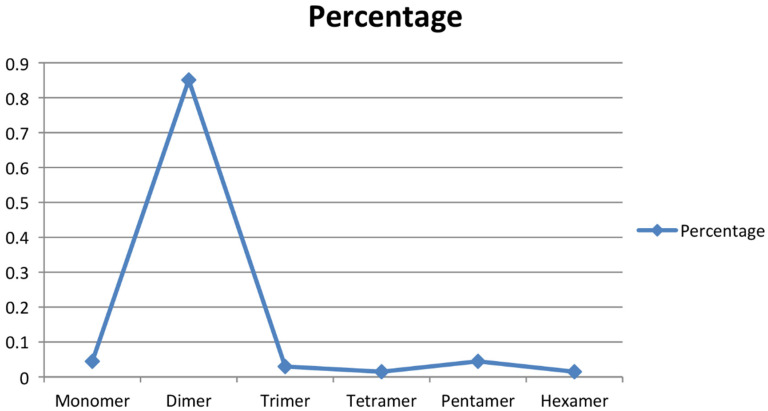
SSR motif-length groups and their percentage occurrence.

To obtain the microsatellite-enriched libraries, the genomic DNA of *A. paniculata* (IC 111291) was digested with restriction enzyme *Sau3A1*, which was further enriched with four types of 3′ biotinylated oligonucleotide probes ((CT)_14_, (GT)_12_, (AG)_15_ and (AAC)_8_). Altogether, 183 recombinant colonies were screened for the presence of CT, GT, AG, and AAC repeats, of which 119 were confirmed as positive clones (65%) through colony PCR and submitted for Sanger sequencing. The SSR finder tool was used to identify the perfect SSR markers, and 47 positive clones (39%) with perfect microsatellite repeats were identified. It was noticed that a few positive clones had more than one microsatellite repeat, and thus, a total of 67 primer pairs were developed (Table 2). The developed and synthesized microsatellite markers had motif-length groups, varying from monomer to hexamer, and their occurrence percentage varied from 1.49 to 85.07 (Figure 2). The tetramer and hexamer motif-length groups had a 1.49% occurrence; trimer had 2.98%, monomer and pentamer had 4.47%, while the dimer motif-length group had a maximum occurrence of 85.07%. Earlier, Wee et al. [33] sequenced 192 clones, and 102 colonies were obtained with desirable SSRs. Furthermore, Kaliswamy et al. [34] also reported that di- and trinucleotide repeats had more occurrences in the *Acanthaceae* family, which is similar to our findings. In addition to that, Lagercrantz et al. [35] and La Rota et al. [36] noticed that GA/CT microsatellite motifs are more abundant than the CA/GT motif in the plant species, which is similar to the present investigation. Marker-assisted breeding essentially requires a robust and informative marker system in the crop of interest [37]. Microsatellite markers are one of the choicest marker systems in molecular breeding of crop species due to its versatile applications in crop genetics and breeding, including cultivar identification [38], genetic diversity assessment [39], genetic mapping [40], gene tagging [41], gene flow [42], and molecular evolution studies [43] on plant species. In *A. paniculate*, the availability of microsatellite markers is lacking, which is a major limitation for its marker-assisted breeding. The screening of microsatellite-enriched libraries and the sequencing of microsatellite-positive clones are effective methods for the development of SSR markers [44].

### 3.2. Validation of g-SSR Loci and Genetic Diversity Statistics

A genetic diversity study among 42 *A. paniculata* accessions was performed using the 67 genomic SSR loci developed from four microsatellite-enriched libraries. The developed g-SSRs were screened for their amplification among the *A. paniculata* accessions, out of which 41 SSRs were found to be polymorphic (Table 3). These SSRs had substantial variations in allele number, which ranged from 2 to 8 with an average of 3.95 alleles per locus, and allele sizes, which ranged from 100 to 870 bp (Figures S4–S6). Similarly, Geng et al. [45] also recorded a range in the number of alleles, from 2 to 8, in *Acanthus ilicifolius*, which is congruent with our results. The PIC value varied from 0.09 for primer Ando4-36-2 to 0.38 for primer Ando5-29, with an average of 0.32. The observed heterozygosity was calculated as 0.00 for several markers, and the highest value was 0.21 for the marker Ando5-12-1, with a mean value of 0.02. Gene diversity (expected heterozygosity) ranged from 0.10 (Ando4-36-2) to 0.50 (Ando5-29, Ando5-26-2, Ando5-14-2, Ando4-27-2, Ando4-26, and Ando2-31-2), with a mean value of 0.40. Similarly, Geng et al. [45] calculated the observed and expected heterozygosity in *Acanthus ilicifolius* using SSR markers, which ranged from 0.200 to 0.875 and 0.227 to 0.798, respectively. Furthermore, Suárez-Montes et al. [46] also calculated the observed and expected heterozygosity values among the *Aphelandra aurantiaca* genotypes, ranging from 0.22 to 0.96 and 0.20 to 0.87, respectively, which is higher than the present study. The present investigation also deciphered large differences between the observed and expected heterozygosity, which indicates that the selected population of *A. paniculata* deviates from Hardy Weinberg’s equilibrium, which might be due to inbreeding, population bottleneck, or random genetic drift [17,45].

### 3.3. Cluster Analysis

**Figure 3:**
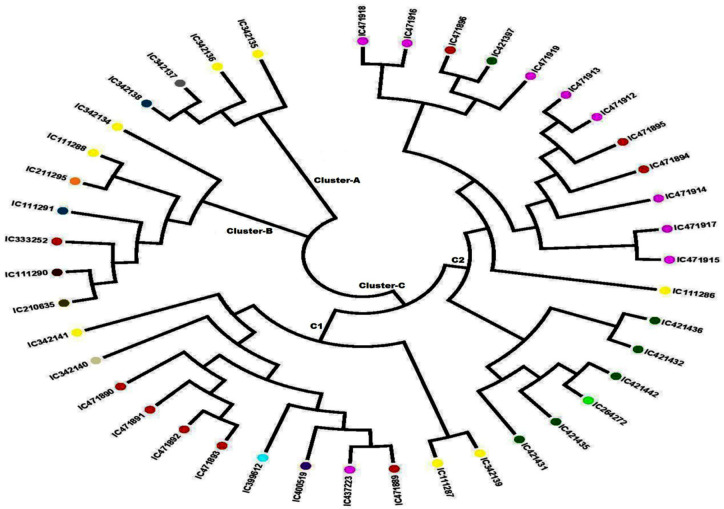
Neighbor-joining (N-J) phylogenetic tree showing the grouping of 42 accessions of *Andrographis paniculata* based on the data of 41 SSR markers.

The unrooted N-J tree was constructed based on 41 developed SSR loci, which clustered all the 42 *A. paniculata* accessions into three major clusters (Figure 3). Earlier, Wijarat et al. [17] clustered 58 *A. paniculata* accessions into two major clusters using SSR markers that are lower than our findings. The genetic distance between the *A. paniculata* accessions ranged from 0.010–0.810, with an average of 0.400. The minimum genetic distance (0.010) was estimated between accessions IC 111291 & IC 211295 and IC 412436 & IC 421432, while the maximum (0.810) was between IC 471917 & IC 471891. Cluster A contained four accessions of *A. paniculata*, out of which two samples were from Uttar Pradesh, one each from Kerala, and one from Maharashtra. Cluster B constituted seven individuals of *A. paniculata*, out of which two were from Uttar Pradesh and one sample each from Andhra Pradesh, Kerala, Madhya Pradesh, Assam, and Tamil Nadu. Cluster C was further divided into two subclusters; subcluster C-1 contained 12 individuals, while C-2 had 19 individuals. Subcluster C-1 showed a tight grouping of four samples from Madhya Pradesh (IC 471890, IC 471891, IC 471892, and IC 471893) and two samples from Uttar Pradesh (IC 111287 and IC 342139). In subcluster C-2, there was a close grouping of two genotypes collected from Chhattisgarh (IC 421436 and IC 421432), and three tight groups from Himachal Pradesh were observed (Tight Group 1—IC 471,915 and IC 471917, Tight Group 2—IC 471912 and IC 471913, Tight Group 3—IC 471916 and IC 471918). Furthermore, *A. paniculate* accessions *viz.* IC 471890, IC 471891, IC 471892, and IC 471893 were in Cluster C-1 while IC 421431, IC 421435, IC 264272, IC 421442, IC 421432 and IC 421436 were in Cluster C2, grouped according to their natural habitat, which is possibly due to less human intervention in their natural habitats. Thus, a few of the *A. paniculate* accessions were tightly grouped according to their habitat and agro-geographical regions, while most of the accessions did not group according to their habitat and agro-geographical regions, which might be due to gene flow in the form of either gamete or genotype.

### 3.4. Population Structure

**Figure 4:**
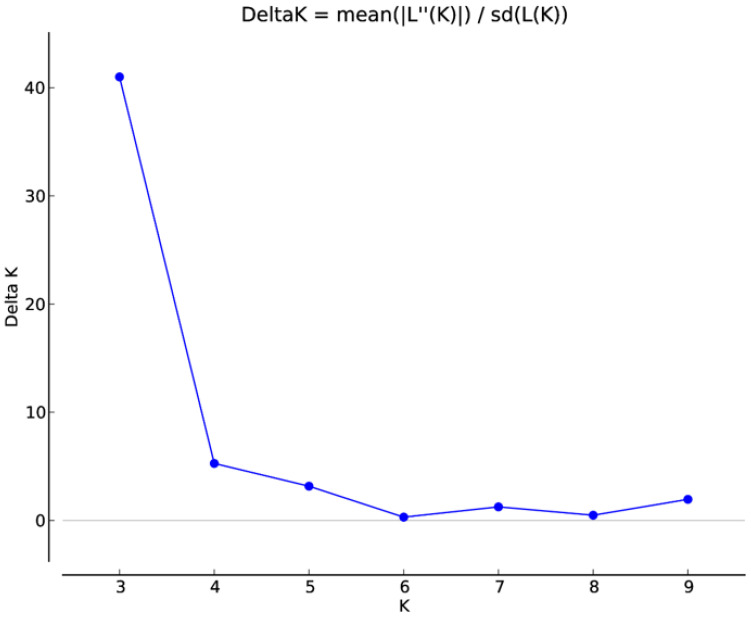
Estimation of the number of populations using LnP(D)-derived Delta k from 2 to 10 using SSR data.

**Figure 5:**
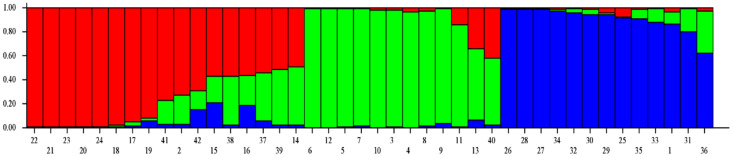
Model-based population structure analysis of 42 accessions of *Andrographis paniculata*.

Model-based population structure analysis was utilized to rebuild the genetic relationship among 42 *A. paniculata* accessions using 41 developed SSR markers. Structure Harvester identified three genetic populations in the present set of *A. paniculate* accessions (Figure 4, Figure 5, Figures S1 and S2). The individuals with a probability score of more than 0.80 are considered genetically pure accession, while a score of <0.80, as an admixture accession. Population I showed eight pure accessions (IC471891, IC 471890, IC 471892, IC 471889, IC 471893, IC 400519, IC 399612, IC 437223) and nine admixed accessions (IC 421442, IC 111287, IC 264272, IC 342140, IC 421432, IC 342141, IC 421431, IC 421435, IC 342139). Population II showed ten pure accessions (IC 210635, IC 342137, IC 111291, IC 211295, IC 342135, IC 111288, IC 111290, IC 333252, IC 342134, IC 342136) and two admixed accessions (IC 342138, IC 421436). Population III showed twelve pure accessions (IC 471895, IC 471913, IC 471912, IC 471919, IC 471917, IC 471915, IC 471914, IC 471894, IC 471896, IC 471918, IC 111286, IC 471916) and one admixed accession (IC 421397). The genetic population differentiation of plant species is the consequence of various processes such as mating strategies, selection, mutations, and gene flow [47]. The genetic population differentiation among the *A. paniculata* accessions might be due to their mating behavior since the crop is self-pollinated and up to 4% cross-pollination occurs through insects [4,17]. The mean Fst value of Population I, Population II, and Population III were 0.4847, 0.5563, and 0.5090, respectively, and the mean alpha value was 0.1075 (Table S2). The allele-frequency divergence between Population I and Population II, Populations II and III, and Populations I and III were 0.2288, 0.1639, and 0.2277, respectively (Table S3). The population structure study indicated the genetic differentiation of *A. paniculata* accessions, which amply suggested that the developed gSSR markers were suitable for population structure studies.

### 3.5. AMOVA, PCoA, and Mantel Test

**Figure 6:**
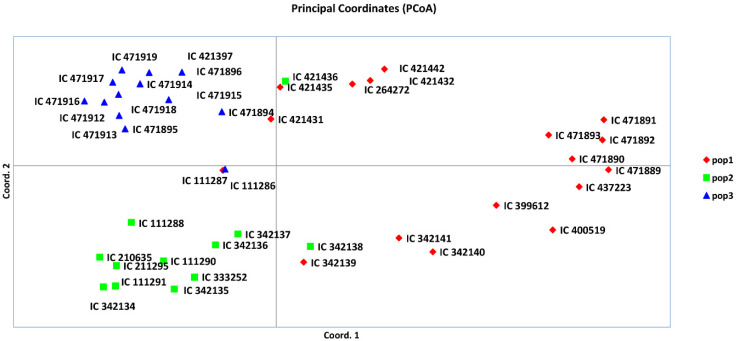
Principal coordinate analysis (PCoA) based on model-based population structure.

An analysis of molecular variance (AMOVA) was undertaken using the three genetic population groups of *A. paniculate*, as deciphered by model-based population structure analysis. The AMOVA illustrated 22% variance among the populations, with 77% variance among the individuals and 1% variance within the individuals of the populations (Table 4 and Figure S3). The first three principal coordinate analyses (PCoA) explained 34.88% cumulative variance, whereas the first, second and third axes explained 14.16%, 11.81%, and 8.91% of genetic variation, respectively (Table S4). Furthermore, the grouping of the *A. paniculata* accessions is depicted in three colors on the coordinates, supplementing the results of the model-based population structure analysis (Figure 6). AMOVA and PCoA explained the substantial genetic diversity among the *A. paniculate* accessions. Furthermore, the Mantel test was performed to obtain the correlation between genetic distance and geographical distance of *A. paniculata* accessions. Overall, a correlation coefficient with a low value (Rxy = 0.046) was observed (Table S5, Figure S7), indicating very little correlation between the genetic and geographical distances of *A. paniculata* accessions [48]. This might be due to the gene flow of *A. paniculata* accessions, in the form of either genotype or gamete.

## 4. Conclusions

Based on this study, it can be concluded that the novel set of g-SSR primer pairs developed in the present study were found to be efficient for molecular characterization of *A. paniculate* accessions. Thus, it can be added as new genomic resources for *A. paniculata* and further utilized in germplasm management and basic population genetics and plant-breeding studies.

## Figures and Tables

**Table 1:** The details of *Andrographis paniculata* accessions studied.

Sl.No.	Accession No.	Habitat	Collection Site	Collection Year	Agro Ecological Regions
1	IC 111286	Disturbed	Haidargarh, Faizabad, Uttar Pradesh	1992	Gangatic Plains
2	IC 111287	Disturbed	Katarnia Forest, Gonda, Uttar Pradesh	1992	Gangatic Plains
3	IC 111288	Natural	BastiForest, Basti, Uttar Pradesh	1992	Gangatic Plains
4	IC 111290	Cultivated	Regional Research Lab (RRL), Jorhat, Assam	1992	N.E. Region
5	IC 111291	Disturbed	Shola Forest, Thiruananthapuram, Kerala	1992	Western Ghats
6	IC 210635	Cultivated	Garden, Namakkal, Tamil Nadu	1997	Eastern Ghats
7	IC 211295	Natural	Ananthagiri Forest, Visakhapatnam, Andhra Pradesh	1997	Eastern Ghats
8	IC 333252	Disturbed	Jamli, Barwani, Madhya Pradesh	1999	Eastern Ghats
9	IC 342134	Natural	Suparia Forest, Bahraich, Uttar Pradesh	1995	Gangatic Plains
10	IC 342135	Natural	Satrikh Forest, Barbanki, Uttar Pradesh	1995	Gangatic Plains
11	IC 342136	Cultivated	Garden, Saharanpur, Uttar Pradesh	1994	Gangatic Plains
12	IC342137	Cultivated	Regional Research Centre (RRC), Pune, Maharashtra	1995	Western Ghats
13	IC 342138	Cultivated	Regional Research Centre (RRC), Thiruananthapuram, Kerala	1995	Western Ghats
14	IC 342139	Cultivated	Regional Research Centre (RRC), Jhansi, Uttar Pradesh	1995	Gangatic Plains
15	IC 342140	Cultivated	Garden, RRC-Kolkata, West Bangal	1995	N. E. Region
16	IC 342141	Cultivated	Narendra Dev University, Faizabad, Uttar Pradesh	1995	Gangatic Plains
17	IC 399612	Natural	Kashidih Forest, East Singhbhum, Jharkhand	1999	Gangatic Plains
18	IC 400519	Cultivated	Adumalleshwaram, Chitradurg, Karnataka	2001	Western Ghats
19	IC 437223	Cultivated	YS Parmar University, Solan, Himachal Pradesh	2003	Western Himalaya
20	IC 471889	Natural	Dindori Forest, Amarkantak, Madhya Pradesh	2003	Eastern Ghats
21	IC 471890	Natural	Dindori Forest, Amarkantak, Madhya Pradesh	2003	Eastern Ghats
22	IC 471891	Natural	Dindori Forest, Amarkantak, Madhya Pradesh	2003	Eastern Ghats
23	IC 471892	Natural	Dindori Forest, Amarkantak, Madhya Pradesh	2003	Eastern Ghats
24	IC 471893	Natural	Dindori Forest, Amarkantak, Madhya Pradesh	2003	Eastern Ghats
25	IC 471894	Natural	Dindori Forest, Amarkantak, Madhya Pradesh	2003	Eastern Ghats
26	IC 471895	Natural	Dindori Forest, Amarkantak, Madhya Pradesh	2003	Eastern Ghats
27	IC 471912	Disturbed	Nauni Forest, Solan, Himachal Pradesh	2002	Western Himalaya
28	IC 471913	Disturbed	Nauni Forest, Solan, Himachal Pradesh	2002	Western Himalaya
29	IC 471914	Disturbed	Ochghat, Solan, Himachal Pradesh	2002	Western Himalaya
30	IC 471915	Disturbed	BatalGhatti, Solan, Himachal Pradesh	2002	Western Himalaya
31	IC 471916	Disturbed	Ochghat, Solan, Himachal Pradesh	2002	Western Himalaya
32	IC 471917	Disturbed	Kunihar, Solan, Himachal Pradesh	2002	Western Himalaya
33	IC 471918	Disturbed	BatalGhatti, Solan, Himachal Pradesh	2002	Western Himalaya
34	IC 471919	Disturbed	BatalGhatti, Solan, Himachal Pradesh	2002	Western Himalaya
35	IC 471896	Natural	Mandla Forest, Mandla, Madhya Pradesh	2003	Eastern Ghats
36	IC 421397	Natural	Machkot, Bastar, Chhattisgarh	2001	Eastern Ghats
37	IC 421431	Natural	Barasur Forest, Dantewada, Chhattisgarh	2001	Eastern Ghats
38	IC 421432	Natural	Barasur Forest, Dantewada, Chhattisgarh	2001	Eastern Ghats
39	IC 421435	Natural	Greedam, Dantewada, Chhattisgarh	2001	Eastern Ghats
40	IC 421436	Natural	Muchnar, Dantewada, Chhattisgarh	2001	Eastern Ghats
41	IC 421442	Natural	Dantewara Forest, Dantewada, Chhattisgarh	2001	Eastern Ghats
42	IC 264272	Natural	Ishapur, Dalsinghsarai, Bihar	2001	Gangatic Plains

**Table 2:** Details of 67 novel genomic simple sequence repeat (SSR) loci developed through microsatellite-enriched genomic libraries.

S.No	Primer ID	Forward Primer Sequence (5′-3′)	Reverse Primer Sequence (5′-3′)	Repeat Motif	Expected Product Size (bp)
1	Ando4-2	CTCTCTCTCGCAGCTCTCTCTC	CTTCGGATCAGTTAGCCCCT	(CT)7	386
2	Ando6-3	AGCTTCGGATCAGTTAGTCCCT	GCTCCCTCTCAGTGTCTCTCTC	(AG)6	378
3	Ando2-3-2	GCTTCGGATCAGTTAGTCCCTT	AGCTCTCTCTCGCAGCTCTCT	(AG)6	388
4	Ando2-3-3	GAGAGACCTGCGAGAGAGAGAG	GAACCAGGCAGAACCAATAATC	(GA)6	241
5	Ando2-6-2	GCTTCGGATCAGTTAGTCCCTT	CTCTCTCTCGCAGCTCTCTCTC	(GA)7	387
6	Ando2-9	GATTATGTGGGAATCTTGGGTG	ATATAGGTGGGCGATAAACCG	(A)15	228
7	Ando2-12	AACAAGGTTACACTCTCCGACC	CTCGATCCTATTCAGTTCCACC	(A)13	388
8	Ando2-23	TTCTTTTCTGTGTAATCGTCGC	CTAAGCGTTGCTCCATTTCTTC	(A)10	188
9	Ando2-24-3	CGGCTCTCTCTCAGTCTCTCTC	CTTCGGGTCAGTTAGTCCCTT	(CT)7	375
10	Ando2-30-2	AGCTTCGGATCAGTTAGTCCCT	CGCTCGTAGTCTCTCTCTCACA	(AG)6	258
11	Ando2-31-2	ATTGATGCCCAAAGAGAAGAAG	CTCTCCCTATCTCGCACTATCG	(AG)6	250
12	Ando2-32-2	AGCTTCGGATCAGTTAGTCCCT	CAGCTCTCCCTCAGTCTCTCTC	(AG)6	378
13	Ando2-40-2	CTCTCTCTCTCTCTCCACAGCC	ATGACCCTCAACATAGCGTTTT	(TC)18	316
14	Ando 4-2-1	ATGACCCTCAACATAGCGTTTT	CTCTCTCTCTCTCTCCACAGCC	(GA)13	318
15	Ando4-3-2	AGCTTCGGATCTCTCTCCACT	ATGACCCTCAACATAGCGTTTT	(TC)9	383
16	Ando4-4-2	CTTCGGGTCAGTTAGTCCCTT	CAGCTCCCTCTCAGTCTCTCTC	(AG)6	374
17	Ando4-11-2	AGCTTCGGATCTCTCTCCACT	ATGACCCTCAACATAGCGTTTT	(TC)9	379
18	Ando2-3	AGCTTCGGATCAGTTAGTCCCT	TCTCTATCTCGCATTCTCTCCC	(AG)6	478
19	Ando2-21	GCCCAAAGAGAAATAGCTGAGA	CTATGACCATGATTACGCCAAG	(GA)6	298
20	Ando2-30-1	GCTTCGGATCAGTTAGTCCCTT	CGCAGCTCTCTCTCAGTCTCTC	(AG)6	381
21	Ando2-30-3	AGCTTCGGATCAGTTAGTCCCT	TATCTCGCACTCTCTCTCTGGC	(GA)7	479
22	Ando2-31-1	CTTCGGATCGGTTAGTCCCT	CTCTCTCTCGCAGCTCTCTCTC	(AG)6	390
23	Ando2-31-3	CTTCGGATCGGTTAGTCCCT	TATCTCGCACTCTCTCTCTGGC	(GA)7	479
24	Ando4-4-1	CTTCGGGTCAGTTAGTCCCTT	CAGCTCCCTCTCAGTCTCTCTC	(AG)6	374
25	Ando4-4-3	CTTCGGGTCAGTTAGTCCCTT	TCTCTATCTCGCACTCTCTCCC	(GA)7	477
26	Ando4-9	CCAGTCCTTTTCTGCTGTTACC	AGCTTCGATCAATTTCCAAGG	(AG)10	173
27	Ando4-9-2	GCTTCGGATCAAAATACTCAGC	CTCTCTTTATGGCCTATCCCCT	(AGGGAG)5	298
28	Ando4-21	AGCTTCGGATCTCTCTCCACT	ATGACCCTCAACATAGCGTTTT	(TC)12	383
29	Ando4-26	AGCTTCGGATCGTAGGGTTT	TCTGTATGTGTGCTCAACCTCC	(TC)14	235
30	Ando4-27	ATGACCCTCAACATAGCGTTTT	CTCTCTCTCTCTCTCCACAGCC	(GA)19	318
31	Ando4-27-2	ATGACCCTCAACATAGCGTTTT	AGCTTCGGATCTCTCTCCACT	(GA)13	385
32	Ando4-31	AGCTTCGGATCAGTTAGTCCCT	TTTCCCTCTCTATCTCGCACTC	(AG)6	489
33	Ando4-32	AGCTTCGGATCAGTTAGTCCCT	GCAGTCTCTCTCGCAACTCTCT	(AG)6	449
34	Ando4-32-1	AGCTTCGGATCAGTTAGTCCCT	TGCAGCTCTCTCTCTCTCAGTTT	(GA)6	499
35	Ando4-34-1	CTCTCTCTCTCTCTCCACAGCC	CAACCTCCATCATCTGAACAAA	(TC)18	253
36	Ando4-34-2	AGCTTCGGATCTCTCTCCACT	ATGACCCTCAACATAGCGTTTT	(TC)12	385
37	Ando4-35-1	CAACCTCCATCATCTGAACAAA	CTCTCTCTCTCTCTCCACAGCC	(GA)19	251
38	Ando4-35-2	ATGACCCTCAACATAGCGTTTT	AGCTTCGGATCTCTCTCCACT	(GA)13	385
39	Ando4-36	AGCTTCGGATCAGTTAGTCCCT	TATCTCGCACTCTCTCTCTGGC	(GA)6	471
40	Ando4-36-2	AGCGATAGTGCGTGATAGGG	GGCCTCTCTCAGTTACAGTCTCC	(GA)6	276
41	Ando4-39	AGCTTCGGATCGTAGGGTTT	TCTGTATGTGTGCTCAACCTCC	(TC)13	233
42	Ando4-41	CTTCGGGTCAGTTAGTCCCTT	TCTCTATCTCGCACTCTCTCCC	(GA)7	479
43	Ando4-42	AATTCCCACAGCAGAGAGAGAG	GTTTCTGACTTTTCACGTTCCC	(GA)14	331
44	Ando4-43/1	CTCTCTCTCTCTCTCCACAGCC	TGACCCTCAACATAGCGTCTTA	(TC)19	317
45	Ando4-43/2	AGCTTCGGATCTCTCTCCACT	TGACCCTCAACATAGCGTCTTA	(TC)12	382
46	Ando5-1	TAACCGAGCATCTCTCTCTGCT	TCAATGGGTATCTGTGTTTTGG	(TCT)4	120
47	Ando5-8	GCTTCGGATCTAACACAACCTC	GAAAAGGGTTCTCCTCCAGTTT	(TCTT)3	187
48	Ando5-10	TTGATGCCCAAAGAGAAATAGC	GTTACAGTCTCCCTTGCAGCTC	(AG)6	487
49	Ando5-12	GAGCGATAGTGCGAGATAGGG	GTTACAGTCTCCCTTGCAGCTC	(GA)8	270
50	Ando5-12-1	CTTCGGATCAGTTAGCCCCT	GTCTTGCACCCACTCTCTCTCT	(GA)6	319
51	Ando5-13	CTCTCTCTCTCTCTCCACAGCC	AAGCGGGATTGATTTACAACAC	(TC)15	388
52	Ando5-13-2	AGCTTCGGATCTCTCTCCACT	AAGCGGGATTGATTTACAACAC	(TC)15	459
53	Ando5-14	AGCTTCGGATCAGTTAGTCCCT	TATCTCGCACTCTCTCTCTGGC	(GA)7	473
54	Ando5-14-2	AGCTTCGGATCAGTTAGTCCCT	CGCACTCTCTCAGTTTTCCTCT	(AG)6	345
55	Ando5-19	GAAGACCCTAATCGAAACATCG	AAAGAACCTCCGCTCATAACAG	(TCTTC)2	264
56	Ando5-23	AGCTTCGGATCAGTTAGTCCCT	GCTCTCTCTCTCGCAGTTTCTC	(AG)6	500
57	Ando5-26	ATTCGGTCATTCTTAGCCCTCT	TCAATGGGTATCTGTGTTTTGG	(TCT)4	158
58	Ando5-26-2	ACCGAGCATCTCTCTCTGCTAT	TTCGGATCTGTCCTGTGTTTC	(AACTC)2	224
59	Ando5-29	CTTCGGATCAGTTAGTCCCTTC	TCTCTATCTCGCAGCTCTCCTT	(GA)6	413
60	Ando5-29-2	AGCTTCGGATCAGTTAGTCCCT	TCTCTCTCTCCCTATCTCGCAC	(AG)6	281
61	Ando5-30	GACAACACATTCCTCAAAAGCC	AGCTTCGGATCTGGTCTAACG	(TC)8	141
62	Ando5-31	CTTCGGGTCAGTTAGTCCCTT	TCCCTCTCTATCTCGCACTCTC	(GA)6	481
63	Ando5-31-2	CGAGCGATAGTGCGTGATA	TCTCCCTCTCCCAGTCTCTC	(GA)6	324
64	Ando5-36	CTTCGGGTCAGTTAGTCCCTT	TCTCTCTCGCAGGTCTCTCTCT	(GA)7	325
65	Ando5-37	CTCCTTGACTATCTTTGGCCTG	TTATGTCTCTGATGATGGGTCG	(TCTTC)2	136
66	Ando5-38	CTCTCTCTCTCTCTCCACAGCC	ATGACCCTCAACATAGCGTTTT	(TC)19	318
67	Ando5-38-2	AGCTTCGGATCTCTCTCCACT	ATGACCCTCAACATAGCGTTTT	(TC)14	387

**Table 3:** Details of allele number, major allele frequency, gene diversity, heterozygosity, and PIC values of developed g-SSRs.

Sl.No.	Primer ID	Ta ^x^(^°^C)	Allele Size Range (bp)	Allele No	Major Allele Frequency	Gene Diversity (Expected Heterozygosity)	Observed Heterozygosity	PIC ^y^
1.	Ando 4-2	45.0	160–170	2	0.81	0.31	0	0.26
2.	Ando 2-24-3	65.4	360–400	4	0.71	0.41	0	0.33
3.	Ando 2-30-2	59.9	270–300	4	0.63	0.45	0	0.35
4.	Ando 4-4-2	60.9	370–390	2	0.76	0.36	0	0.30
5.	Ando 4-3-2	60.9	390–490	6	0.75	0.35	0	0.28
6.	Ando 4-11-2	60.9	390–410	2	0.85	0.26	0	0.22
7.	Ando 4-2-1	60.9	320–360	4	0.70	0.41	0.19	0.33
8.	Ando 4-40-2	62.0	310–320	2	0.71	0.42	0	0.33
9.	Ando 2-32-2	40.9	380–390	2	0.68	0.44	0	0.34
10.	Ando 2-31-2	40.9	250–260	2	0.53	0.50	0	0.37
11.	Ando 2-21	41.9	720-760	2	0.89	0.19	0	0.17
12.	Ando 4-9-2	40.9	780–820	4	0.70	0.42	0	0.33
13.	Ando 4-21	59.3	410–820	4	0.72	0.41	0	0.32
14.	Ando 4-26	50.9	240–250	2	0.54	0.50	0	0.37
15.	Ando 4-27	45.6	310–340	4	0.84	0.25	0	0.21
16.	Ando 4-27-2	43.0	390–400	2	0.52	0.50	0	0.37
17.	Ando 4-31	40.9	290–360	8	0.66	0.41	0	0.32
18.	Ando 4-32	40.4	210–360	6	0.72	0.39	0	0.31
19.	Ando 4-32-1	48.4	360–450	8	0.73	0.36	0	0.29
20.	Ando 4-34-1	54.3	310–320	2	0.69	0.43	0	0.34
21.	Ando 4-34-2	54.3	320–430	8	0.69	0.40	0	0.31
22.	Ando 4-35-1	54.3	220–280	4	0.61	0.47	0.15	0.36
23.	Ando 4-35-2	43.0	320–410	6	0.64	0.45	0	0.35
24.	Ando 4-36-2	43.0	230–340	4	0.95	0.10	0	0.09
25.	Ando 4-39	40.9	140-260	4	0.77	0.36	0.06	0.29
26.	Ando 4-41	40.9	620–680	6	0.66	0.39	0	0.30
27.	Ando 4-43/1	40.4	320–350	4	0.58	0.49	0	0.37
28.	Ando 4-43/2	40.9	390–430	4	0.68	0.41	0	0.32
29.	Ando 5-1	51.9	420–450	4	0.64	0.45	0	0.35
30.	Ando 5-10	40.1	260–490	4	0.66	0.44	0.10	0.35
31.	Ando 5-12	49.3	100–220	8	0.82	0.30	0	0.25
32.	Ando 5-12-1	51.9	370–560	4	0.54	0.49	0.21	0.37
33.	Ando 5-13	58.4	390–420	4	0.76	0.37	0	0.30
34.	Ando 5-13-2	51.9	390–510	4	0.64	0.44	0	0.34
35.	Ando 5.14	51.9	330–360	4	0.66	0.43	0	0.34
36.	Ando 5-14-2	40.4	390–410	2	0.52	0.50	0	0.37
37.	Ando 5-26-2	54.3	850–870	2	0.53	0.50	0	0.37
38.	Ando 5-29	54.3	420–440	2	0.50	0.50	0	0.38
39.	Ando 5-30	40.4	250–350	4	0.65	0.41	0	0.32
40.	Ando 5-31-2	40.4	330–550	4	0.65	0.45	0.12	0.35
41.	Ando 5-37	51.9	210–290	4	0.70	0.41	0	0.33
**Mean**				3.95	0.68	0.40	0.02	0.32

^x^ Ta = annealing temperature. ^y^ PIC = polymorphic information content.

**Table 4:** Summary of analysis of molecular variance of 41 genomic SSRs among 42 Kalmegh accessions.

Source	df	SS	MS	Estimated Variance	%
Among Populations	2	310.537	155.268	4.453	22%
Among Individual	39	1250.952	32.076	15.877	77%
Within Individual	42	13.5	0.321	0.321	1%
Total	83	1574.988		20.651	100%

## Supplementary Materials

The following are available online at https://www.mdpi.com/2223-7747/9/12/1734/s1. Figure S1: Analysis of molecular variance (AMOVA) of 42 accessions of *Andrographis paniculata* based on SSR marker data. Figure S2: Plot of mean likelihood L (K) and variance per K value from STRUCTURE on SSR dataset. Figure S3: Table output of the Evanno method results; yellow highlight is performed dynamically on the Structure Harvester online tool and shows the highest value in the Delta K column. Figure S4: Gel image of primer Ando4-34-1 among the 42 accessions of *A. paniculate*; M = 100 bp marker; polymorphic bands are indicated with a yellow arrow. Figure S5: Gel image of primer Ando2-40-2 among the 42 accessions of *A. paniculata*. Figure S6: Gel image of primer Ando4-26 among the 42 accessions of *A. paniculata*. Figure S7: Relationship between genetic and geographic distances for 42 *A. paniculata* accessions. Table S1: Nanodrop DNA quantification result of 42 *A. paniculata* accessions. Table S2: Mean value of Fst1, Fst2, Fst3, and alpha concluded from a model-based approach. Table S3: Allele-frequency divergence among populations of *A. paniculata* accessions. Table S4: Percentage of variation explained by the first three axes among the *A. paniculata* accessions. Table S5: Mantel results for geographic distance vs. genetic distance.
