# Supplementary material for: Development of Novel Genomic Simple Sequence Repeat (g-SSR) Markers and Their Validation for Genetic Diversity Analyses in Kalmegh [Andrographis paniculata (Burm. F.) Nees]
Source: Plants (Basel). 2020 Dec 9;9(12):1734. doi: 10.3390/plants9121734 (PMC7763852; doi:10.3390/plants9121734)
Supplement: Supplementary file 1 [file plants-09-01734-s001.pdf]

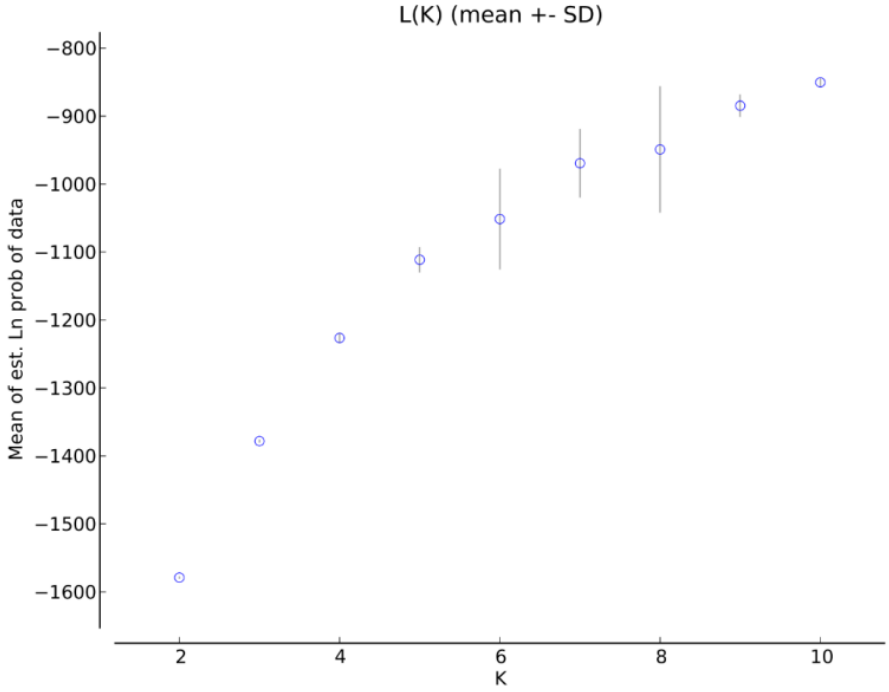
L(K) (mean +- SD)
Mean of est. Ln prob of data
−800
−900
−1000
−1100
−1200
−1300
−1400
−1500
−1600
2
4
6
8
10
K

Figure S1

K	Reps	Mean LnP(K)	Stdev LnP(K)	Ln'(K)	|Ln''(K)|	Delta K
2	5	-1578.980000	0.831865	—	—	—
3	5	-1378.220000	1.194571	200.760000	48.980000	41.002165
4	5	-1226.440000	6.964410	151.780000	36.680000	5.266778
5	5	-1111.340000	17.424925	115.100000	55.120000	3.163285
6	5	-1051.360000	73.038640	59.980000	22.040000	0.301758
7	5	-969.340000	49.292525	82.020000	61.680000	1.251305
8	5	-949.000000	91.795316	20.340000	43.980000	0.479109
9	5	-884.680000	15.344119	64.320000	29.880000	1.947326
10	5	-850.240000	4.715188	34.440000	—	—

Figure S2

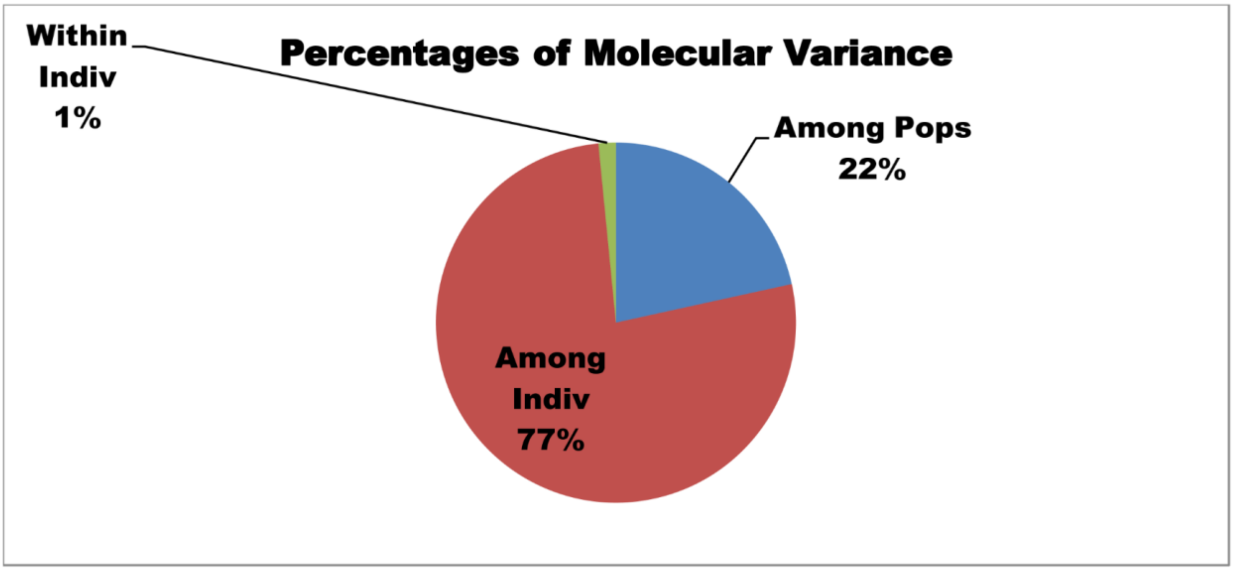
Percentages of Molecular Variance
Within Indiv 1%
Among Pops 22%
Among Indiv 77%

Figure S3

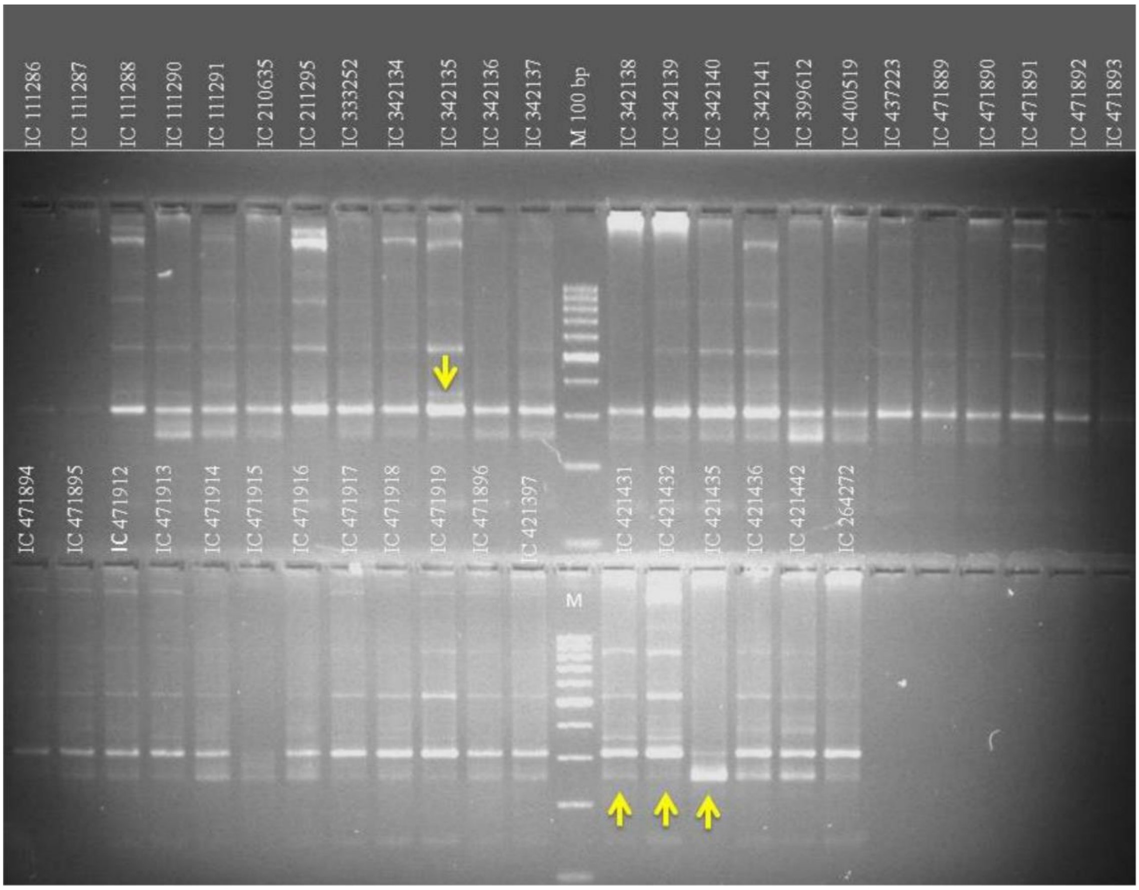
IC 111286
IC 111287
IC 111288
IC 111290
IC 111291
IC 210635
IC 211295
IC 333252
IC 342134
IC 342135
IC 342136
IC 342137
M 100 bp
IC 342138
IC 342139
IC 342140
IC 342141
IC 399612
IC 400519
IC 437223
IC 471889
IC 471890
IC 471891
IC 471892
IC 471893
IC 471894
IC 471895
IC 471912
IC 471913
IC 471914
IC 471915
IC 471916
IC 471917
IC 471918
IC 471919
IC 471896
IC 421397
M
IC 421431
IC 421432
IC 421435
IC 421436
IC 421442
IC 264272

Figure S4

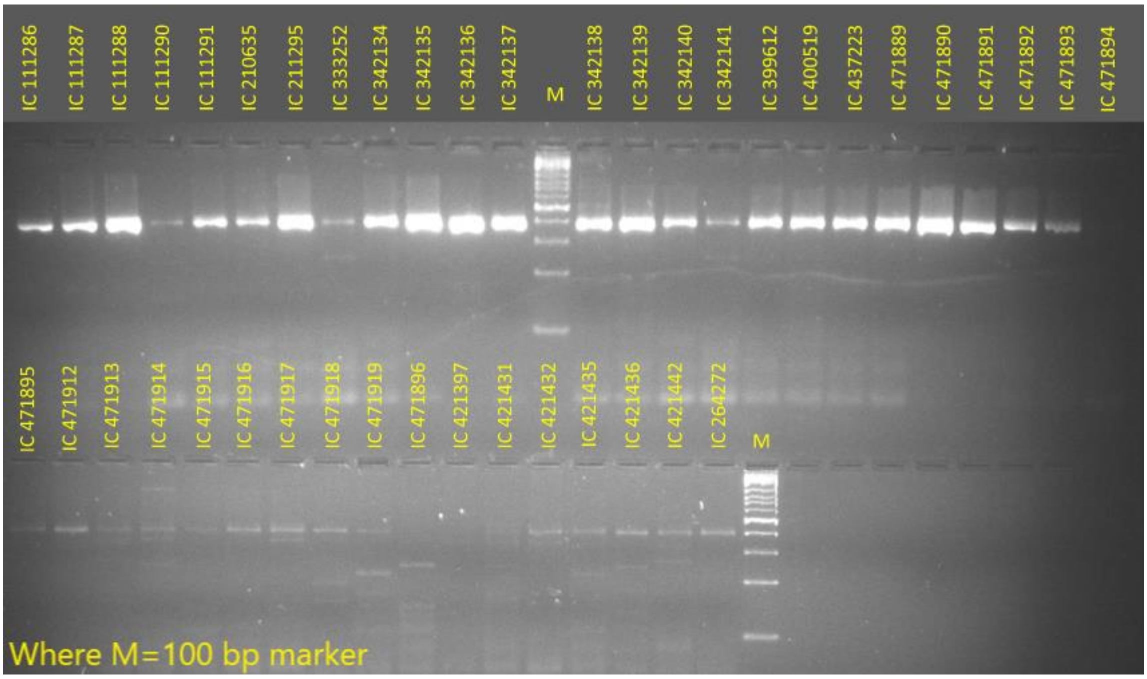
IC 111286
IC 111287
IC 111288
IC 111290
IC 111291
IC 210635
IC 211295
IC 333252
IC 342134
IC 342135
IC 342136
IC 342137
M
IC 342138
IC 342139
IC 342140
IC 342141
IC 399612
IC 400519
IC 437223
IC 471889
IC 471890
IC 471891
IC 471892
IC 471893
IC 471894
IC 471895
IC 471912
IC 471913
IC 471914
IC 471915
IC 471916
IC 471917
IC 471918
IC 471919
IC 471896
IC 421397
IC 421431
IC 421432
IC 421435
IC 421436
IC 421442
IC 264272
M
Where M=100 bp marker

Figure S5

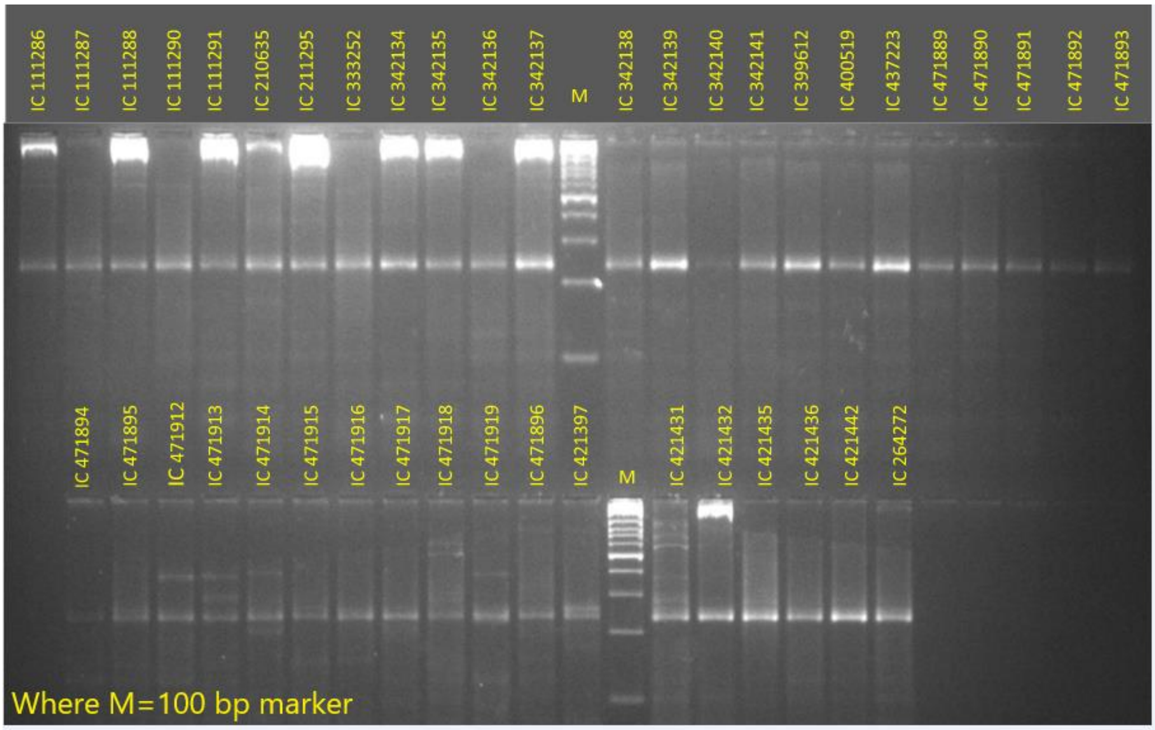
IC 111286
IC 111287
IC 111288
IC 111290
IC 111291
IC 210635
IC 211295
IC 333252
IC 342134
IC 342135
IC 342136
IC 342137
M
IC 342138
IC 342139
IC 342140
IC 342141
IC 399612
IC 400519
IC 437223
IC 471889
IC 471890
IC 471891
IC 471892
IC 471893
IC 471894
IC 471895
IC 471912
IC 471913
IC 471914
IC 471915
IC 471916
IC 471917
IC 471918
IC 471919
IC 471896
IC 421397
M
IC 421431
IC 421432
IC 421435
IC 421436
IC 421442
IC 264272
Where M=100 bp marker

Figure S6

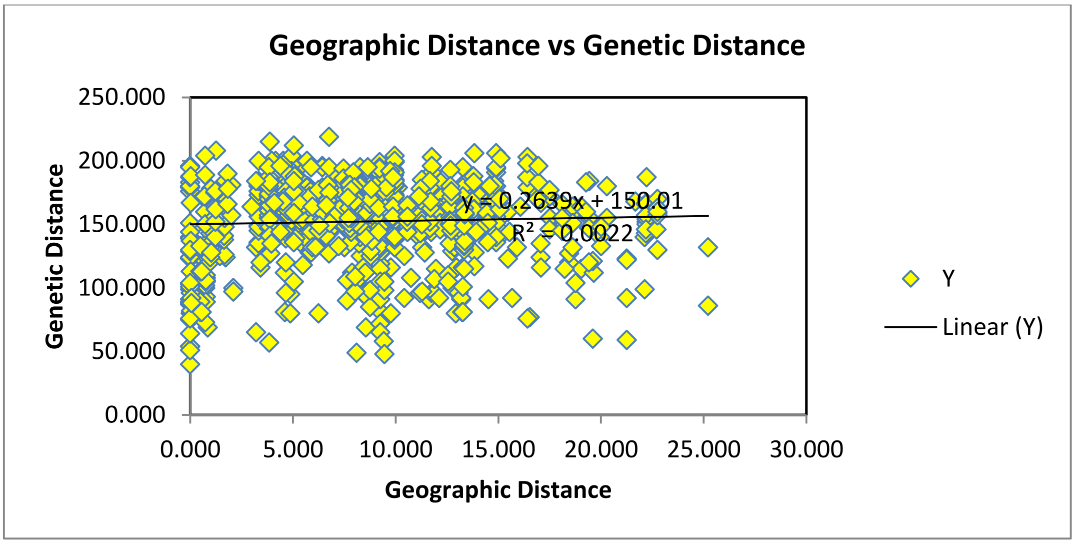
Geographic Distance vs Genetic Distance
Genetic Distance
250.000
200.000
150.000
100.000
50.000
0.000
0.000
5.000
10.000
15.000
20.000
25.000
30.000
Geographic Distance
y = 0.2639x + 150.01
R² = 0.0022
Y
Linear (Y)

Figure S7

Table S1: Nanodrop DNA quantification result of 42 *A. paniculata* accessions:

S.No.	Accession No.	DNA Concentration (ng/ul)	260/280 Ratio	260/230 Ratio
1	IC 111286	478.73	2.08	2.03
2	IC 111287	637.21	1.97	1.91
3	IC 111288	835.34	1.94	1.74
4	IC 111290	362.24	1.91	1.79
5	IC 111291	1194.56	1.76	2.2
6	IC 210635	940.12	1.62	1.9
7	IC 211295	1089.23	1.87	2.2
8	IC 333252	393.46	1.92	1.98
9	IC 342134	467.76	1.9	1.97
10	IC 342135	973.98	1.98	2.01
11	IC 342136	279.23	1.67	1.91
12	IC342137	611.22	1.63	1.92
13	IC 342138	340.16	2	1.98
14	IC 342139	804.87	1.81	1.99
15	IC 342140	203.76	1.63	2.3
16	IC 342141	339.32	1.86	1.95
17	IC 399612	298.48	1.81	2.1
18	IC 400519	940.23	1.67	2.09
19	IC 437223	639.87	1.89	1.68
20	IC 471889	793.37	1.78	1.65
21	IC 471890	786.43	1.73	1.87
22	IC 471891	1030.48	1.84	2.31
23	IC 471892	603.28	1.79	2.23
24	IC 471893	680.83	1.84	1.91
25	IC 471894	590.12	1.71	1.96
26	IC 471895	635.36	1.83	1.89
27	IC 471912	380.65	1.81	1.68
28	IC 471913	784.23	1.77	1.86
29	IC 471914	734.86	1.86	2.09
30	IC 471915	582.64	1.74	1.93
31	IC 471916	1035.23	1.83	1.97
32	IC 471917	215.26	1.83	1.89
33	IC 471918	240.16	1.73	2.19
34	IC 471919	402.63	1.89	1.97
35	IC 471896	393.48	1.68	1.96
36	IC 421397	621.29	1.76	1.99
37	IC 421431	672.49	1.65	1.97
38	IC 421432	648.28	1.95	2.13
39	IC 421435	1073.86	1.61	1.98
40	IC 421436	1004.27	1.71	2.17
41	IC 421442	203.65	1.66	1.95
42	IC 264272	648.24	1.73	1.91

Table S2: Mean value of Fst1, Fst2, Fst3 and alpha concluded from model-based approach.

**Mean value of Fst_1**	**0.4847**
**Mean value of Fst_2**	0.5563
**Mean value of Fst_3**	0.5090
**Mean value of alpha**	0.1075

Table S3: Allele-frequency divergence among populations of *Kalmegh* genotypes.

	Pop 1	Pop 2	Pop 3
**Pop 1**	-	0.2288	0.2277
**Pop 2**	0.2288	-	0.1639
**Pop 3**	0.2277	0.1639	-

Table S4: Percentage of variation explained by the first 3 axes among the *Kalmegh* accessions.

**Axis**	**1**	**2**	**3**
Variation (%)	14.16	11.81	8.91
Cumulative variation (%)	14.16	25.97	34.88

Table S5: Mantel Results for geographic distance vs genetic distance

SSx	SSy	SPxy	Rxy	P(rxy-rand >= rxy-data)
25606.107	828401.542	6757.467	0.046	0.150
